# From Command-Control to Lifecycle Regulation: Balancing Innovation and Safety in China’s Pharmaceutical Legislation

**DOI:** 10.3390/healthcare13060588

**Published:** 2025-03-07

**Authors:** Jing Zhang, Shuchen Tang, Pengqing Sun

**Affiliations:** School of Law, Tsinghua University, Beijing 100084, China; zhangjing1016@mail.tsinghua.edu.cn (J.Z.);

**Keywords:** pharmaceutical regulation, lifecycle management, China’s Drug Administration Law, innovation–safety balance, global governance

## Abstract

**Background:** China’s pharmaceutical regulatory framework is undergoing a pivotal shift from a traditional “command-control” model to a “lifecycle regulation” approach, aiming to balance drug safety, innovation, and accessibility. This study systematically examines the evolution, achievements, and challenges of China’s regulatory reforms, offering insights for global pharmaceutical governance. **Methods:** Using a mixed-methods approach integrating historical analysis, policy text mining, and case studies, we reviewed the pharmaceutical laws and regulations enacted since 1949, supplemented by case studies (e.g., COVID-19 vaccine emergency approvals) and a comparative analysis with international models (e.g., U.S. FDA and EU EMA frameworks). The data were sourced from authoritative platforms such as the PKULAW database, criminal law amendments, and international regulatory texts. **Results:** China’s regulatory evolution is categorized into four phases: Emergence (1949–1984), Foundational (1985–2000), Deepening Reform (2001–2018), and Lifecycle Regulation (2019–present). The revised Drug Administration Law (2019) institutionalized risk management, dynamic GMP inspections, and post-market surveillance, marking a transition to holistic lifecycle oversight. Key milestones include the introduction of the Vaccine Management Law (2019) and stricter penalties under the Criminal Law Amendment (XI) (2020). **Conclusions:** China’s lifecycle regulation model demonstrates potential to harmonize safety and innovation, evidenced by improved API export compliance (e.g., 15% increase in international certifications by 2023) and accelerated approvals for breakthrough therapies (e.g., domestically developed PD-1 inhibitors). However, challenges persist, including uneven enforcement capacities, tensions between conditional approvals and risk mitigation, and reliance on global supply chains. These findings provide critical lessons for developing countries navigating similar regulatory dilemmas.

## 1. Introduction

Pharmaceutical regulation is a critical determinant of public health outcomes [1], tasked with ensuring drug safety [2], efficacy, and equitable access while fostering innovation to address unmet medical needs [3]. As the world’s largest producer of active pharmaceutical ingredients (APIs) and the second-largest pharmaceutical market [4], China’s regulatory reforms hold significant implications for global health security and supply chain resilience [5]. Historically, China’s regulatory framework relied on a rigid “command-control” model [6], with a primary focus on the pre-market phase of drug regulation. Centralized administrative oversight prioritized pre-market approval, while post-market regulation was largely neglected [7]. This approach, though effective in standardizing production during early industrialization [8], led to the following systemic vulnerabilities: inconsistent drug quality due to fragmented enforcement [9], delayed innovation from bureaucratic bottlenecks [10], and recurring supply chain disruptions [11]. These challenges mirror global dilemmas, exemplified by India’s 2022 WHO-flagged contaminated cough syrup which was linked to pediatric fatalities in Uzbekistan [12], and the U.S.’s record 323 drug shortages in 2024, driven by inflexible approval processes that stifle market agility [13]. Globally, regulatory paradigms are shifting toward lifecycle governance [14], integrating risk-based evaluations [15], real-time pharmacovigilance [16], and cross-border collaboration [17]. The European Medicines Agency’s (EMA) adaptive pathways for orphan drugs [18], the U.S. FDA’s Sentinel Initiative for post-market monitoring [19], and the International Council for Harmonisation (ICH) guidelines emphasize proactive risk management over reactive compliance [20]. India has integrated supplier and supply chain information to create a digital regulatory system [21]. Brazil’s ANVISA has reinforced the traceability of pharmaceuticals [22].

Aligning with these trends, China’s 2019 revision of the Drug Administration Law marked a watershed moment, institutionalizing lifecycle regulation to address systemic gaps [23]. This model ensures the comprehensive and seamless oversight of pharmaceuticals, covering both pre-market and post-market stages. Key innovations included dynamic Good Manufacturing Practice (GMP) inspections replacing static certifications, risk-proportionate clinical trial approvals [24], and enhanced penalties under the Criminal Law Amendment (XI) (2020) [25]. These reforms not only harmonized domestic practices with global standards—evidenced by a 15% surge in API international certifications (2021–2023) [26]—but also tailored solutions to local priorities, such as the Vaccine Management Law (2019) [27], which established end-to-end oversight for COVID-19 vaccines [28]. For instance, China’s rapid emergency use authorization (EUA) for domestically developed mRNA vaccines during the pandemic underscored the agility of its reformed regulatory framework [29].

Despite growing academic interest in regulatory modernization, existing studies remain disproportionately focused on Western models [30], with limited systematic analysis of China’s hybrid approach [31]. This gap is critical given China’s dual role as a global API supplier [32] and a burgeoning hub for biopharmaceutical innovation [33]. This study addresses this lacuna by interrogating China’s regulatory evolution through four phases: the Emergence era (1949–1984), defined by reactive narcotics control via administrative mandates; the Foundational phase (1985–2000), which criminalized counterfeit drugs and codified production standards; the Deepening Reform period (2001–2018), catalyzed by scandals like the 2016 Changsheng substandard vaccine crisis; and the Lifecycle Regulation era (2019–present), characterized by proactive oversight spanning from R&D to post-market pharmacovigilance. By analyzing 200+ legal texts from the PKULAW database, case studies (e.g., accelerated approvals for PD-1 inhibitors like Camrelizumab [34]), and comparative insights from the U.S. FDA [35] and EU EMA [36], this research elucidates China’s unique trajectory in balancing safety [37], innovation [38], and accessibility [39]—a “regulatory trilemma” framework with implications for low- and middle-income countries (LMICs) [40].

This study advances three key contributions to global health policy. First, it operationalizes lifecycle regulation in a non-Western context, demonstrating how China’s hybrid model—combining centralized mandates with market-driven incentives—addresses the trilemma. Second, it critiques persistent disparities [41], such as rural–urban enforcement gaps and overdependence on imported APIs, which threaten equitable access [5]. Third, it proposes actionable strategies for LMICs, including AI-enhanced compliance tools [42] and regional regulatory harmonization [43], to mitigate similar challenges. By contextualizing China’s reforms within global health governance, this analysis offers pragmatic insights for policymakers striving to reconcile innovation imperatives with public health safeguards in an era of escalating antimicrobial resistance and pandemic risks.

## 2. Materials and Methods

This study employs a mixed-methods approach integrating historical analysis, policy text mining, and case studies to systematically examine the evolution of China’s pharmaceutical regulatory framework. The methodology is designed to align with the research objectives of tracing legislative transitions, evaluating policy efficacy, and comparing domestic practices with international standards.

### 2.1. Data Sources and Collection

The primary data for this study were derived from legal and regulatory texts, case records, and international reference frameworks. Legal documents, including laws, administrative regulations, and judicial interpretations, were systematically retrieved from the PKULAW database (https://www.pkulaw.com/ (24 November 2024)), a comprehensive and authoritative legal information platform in China. To ensure methodological rigor, a dual-phase search strategy was implemented. First, a title-based search using the keyword “药品” (The term “药品” drug in Chinese law broadly refers to pharmaceutical products, including medicines, vaccines, and therapeutic substances, rather than being limited to narcotics or controlled substances. This distinction is critical for accurately interpreting the scope of China’s regulatory framework) identified the core legislation directly governing pharmaceutical regulation, yielding eight laws, 24 administrative regulations, and 11 judicial interpretations. Second, a full-text search expanded the scope to broader regulatory contexts, retrieving 92 laws, 171 administrative regulations, and 44 judicial interpretations. The documents were filtered based on their direct relevance to pharmaceutical administrative supervision and criminal regulations, with non-binding guidelines, duplicates, and unrelated texts (e.g., agricultural or chemical regulations) excluded.

This process resulted in 51 key legal texts, categorized into three groups: (1) administrative legislation, such as revisions of the Drug Administration Law (1984–2019) and its supporting regulations (e.g., Drug Registration Measures, Good Manufacturing Practice); (2) criminal legislation, including provisions from the Criminal Law on drug-related crimes (e.g., Articles 141–142 on counterfeit drugs) and subsequent amendments (e.g., Criminal Law Amendment XI, 2020); and (3) international references, such as U.S. FDA regulations (e.g., Federal Food, Drug, and Cosmetic Act) and EU EMA guidelines, sourced from official government portals and peer-reviewed publications. High-impact examples, such as the Changsheng vaccine scandal (2016) and the emergency approval of COVID-19 vaccines (2020–2022), reflect key regulatory milestones and challenges. These cases are analyzed through the triangulation of court rulings, media reports, and academic critiques. This systematic approach ensures the inclusion of both historical and contemporary materials, providing a solid foundation for analyzing China’s regulatory transformation.

### 2.2. Analytical Framework

This study constructs a multi-dimensional analytical framework to systematically explore China’s transition from a command-control to a lifecycle pharmaceutical regulatory model. The framework integrates historical periodization, policy text deconstruction, case-comparative triangulation, and a theoretical trilemma lens, ensuring empirical rigor and theoretical depth while aligning with global health governance research standards.

China’s regulatory evolution is contextualized through four distinct historical phases, each defined by legislative milestones and socio-political dynamics. The Emergence stage (1949–1984) was characterized by fragmented administrative decrees targeting narcotics control and foundational quality oversight, exemplified by the 1978 Regulations on Narcotics Control. The Foundational stage (1985–2000) saw the codification of the first Drug Administration Law (1985) and specialized regulations such as the Measures for Radioactive Drugs, establishing a legal framework for production and distribution. The Deepening Reform stage (2001–2018) was driven by post-scandal overhauls, including revisions to the Drug Administration Law (2001, 2013, 2015) following crises like the 2016 Changsheng vaccine incident, which exposed systemic enforcement gaps. The Lifecycle Regulation stage (2019–present) emerged with the 2019 Drug Administration Law, institutionalizing holistic oversight from R&D to post-market pharmacovigilance, supported by dynamic Good Manufacturing Practice (GMP) inspections and the Vaccine Management Law (2019).

Policy texts were deconstructed through content coding and thematic mapping using NVivo 12, focusing on recurring terms such as “风险管理” (risk management) and “动态检查” (dynamic inspection). To ensure consistency and clarity, terms with ambiguous meanings, such as “dynamic inspections” versus “risk-based evaluations”, were explicitly defined in the schema. Multiple iterations of coding were conducted to refine the themes, and discrepancies were resolved by consulting legislative commentaries and the relevant academic literature. Legislative impacts were assessed by correlating amendments, such as the enhanced penalties for counterfeit drugs introduced post-2019, with measurable outcomes. One significant metric, for example, was the 15% increase in international certifications for Active Pharmaceutical Ingredients (APIs) between 2021 and 2023, at a CAGR of 7.86% during the forecast period (2025–2030) [44,45,46]. Case studies, including the COVID-19 vaccine emergency approvals (2020–2022) and the Changsheng vaccine scandal (2016), were analyzed to evaluate policy implementation. These case studies were selected for their significance in demonstrating China’s regulatory evolution and the shift towards lifecycle regulation. Data triangulation was employed by incorporating multiple data sources, such as media reports, judicial rulings, and academic critiques, to offer a comprehensive analysis of the policy’s real-world impacts. Additionally, comparative benchmarks against the U.S. FDA and EU EMA frameworks highlighted convergences (e.g., risk-based evaluations) and divergences (e.g., rural enforcement disparities), providing insights into global best practices and areas for improvement in China’s regulatory framework.

Guided by the “safety–innovation–accessibility trilemma” framework, this study examines how China’s reforms balance competing priorities. Safety was enhanced through stricter criminal penalties under the Criminal Law Amendment XI (2020), innovation accelerated via streamlined approvals, and accessibility addressed through API supply chain stabilization. However, tensions still persist, such as conditional approvals raising safety risks. Methodological rigor is ensured through the triangulation of legal texts, case studies, and international benchmarks, complemented by inter-coder reliability tests. The limitations include a reliance on public documents, which may exclude internal policy deliberations, and the partial coverage of regional enforcement gaps. This framework not only elucidates China’s regulatory trajectory but also offers a replicable model for analyzing governance transitions in global health contexts, emphasizing the interplay of legislative intent, practical implementation, and theoretical trade-offs.

## 3. Results

Inter-coder reliability tests yielded a Cohen’s Kappa coefficient of 0.82 (substantial agreement), validating the consistency of legal text interpretation. This methodological rigor supports the analysis of China’s pharmaceutical regulatory evolution across four distinct phases: the Emergence stage (1949–1984), characterized by fragmented narcotics controls; the Foundational stage (1985–2000), establishing production standards through the Drug Administration Law; the Deepening Reform stage (2001–2018), marked by post-scandal legislative tightening; and the Lifecycle Regulation stage (2019–present), marked by transitioning to dynamic oversight and risk management under the 2019 Drug Administration Law. The results reveal a paradigm shift from rigid command-control governance to adaptive lifecycle regulation, balancing safety, innovation, and accessibility while addressing enforcement disparities and global compliance challenges.

### 3.1. Inter-Coder Reliability Tests

To ensure consistency and objectivity in the interpretation of legal and policy texts, inter-coder reliability tests were conducted using Cohen’s Kappa coefficient (K). Two independent coders with expertise in pharmaceutical law and public policy were trained to apply a predefined coding schema, which included thematic categories such as “risk management”, “innovation incentives”, and “post-market surveillance”. A randomly selected subset of 20% of the legal texts (*n* = 10 out of 51) was coded independently by both researchers. The results yielded a Cohen’s K score of 0.82, indicating substantial agreement between the coders (Table 1). Discrepancies primarily arose from ambiguous terminology, such as distinguishing between “dynamic inspections” and “risk-based evaluations”. These differences were resolved through iterative discussions and by referencing legislative intent documents (e.g., official commentaries on the Drug Administration Law). The high inter-coder reliability underscores the robustness of the thematic analysis and minimizes subjective bias in categorizing legislative priorities.

The reform of China’s pharmaceutical management system, as classified above, is a process of gradual development. The pharmaceutical management system is closely related to the socio-economic system, and the characteristics of the pharmaceutical market differ at different stages, leading to varying focus in the pharmaceutical regulation models. This process is mainly divided into four stages: the Emergence stage (1949–1983), the Foundational stage (1984–2000), the Deep Reform stage (2001–2018), and the full Lifecycle Regulation stage (2019 to the present).

### 3.2. Statistical Overview of Legal and Regulatory Documents Across Different Stages

**Figure 1:**
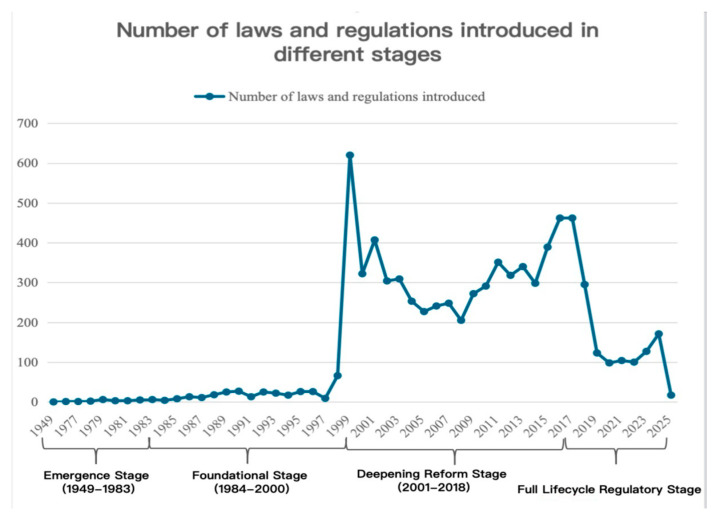
Number of laws and regulations introduced in different stages.

**Figure 2:**
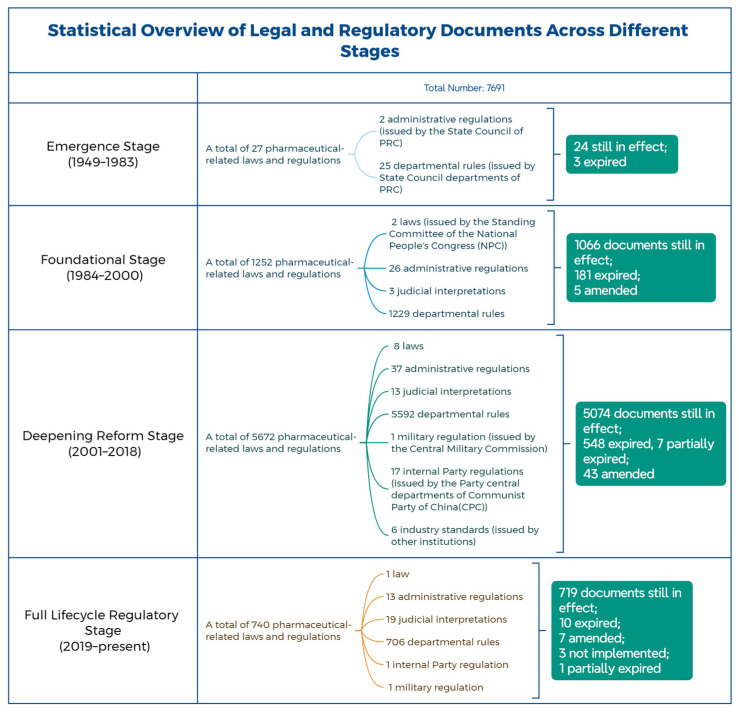
Statistical overview of legal and regulatory documents across different stages.

This study utilizes the legal and regulatory search system of Peking University’s Fahbao website to conduct a statistical analysis of the number and efficacy level of the pharmaceutical management-related laws and regulations introduced during each of the four stages and for each year within those stages, using “pharmaceuticals” as the keyword. The aim is to gain an understanding of the legislative landscape for pharmaceuticals in each period (Figure 1 and Figure 2).

#### 3.2.1. Emergence Stage (1949–1983)

**Figure 3:**
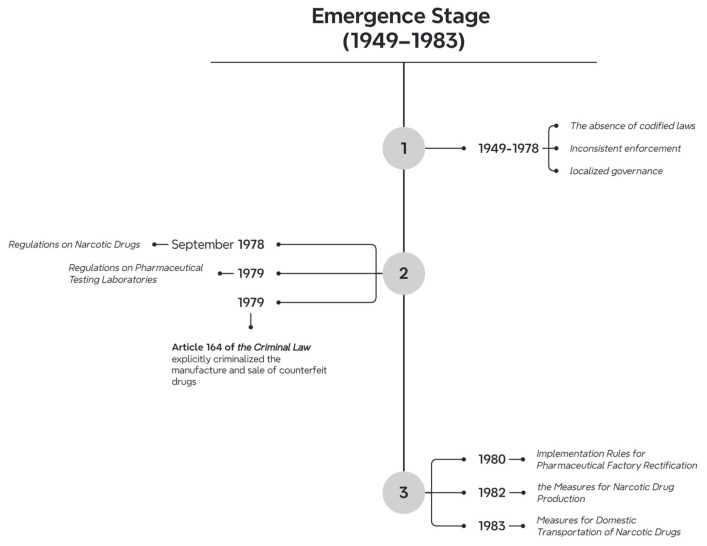
Timeline of legislation during the Emergence stage.

Between 1949 and 1983, a total of 27 pharmaceutical-related laws and regulations were introduced. In terms of the publication years, one document was issued in 1957, one in 1977, two in 1978, six in 1979, three in 1980, three in 1981, five in 1982, and six in 1983. Regarding the hierarchy of legal force, there were two administrative regulations and 25 departmental rules. In terms of the issuing bodies, two documents were issued by the State Council, and the rest were issued by various State Council departments. As for their validity, twenty-four documents are still in effect, while three have expired. The dominant legal document during this period was the Regulations on the Management of Narcotic Drugs, with the others serving as supplementary legal norms (Figure 3).

#### 3.2.2. Foundational Stage (1984–2000)

**Figure 4:**
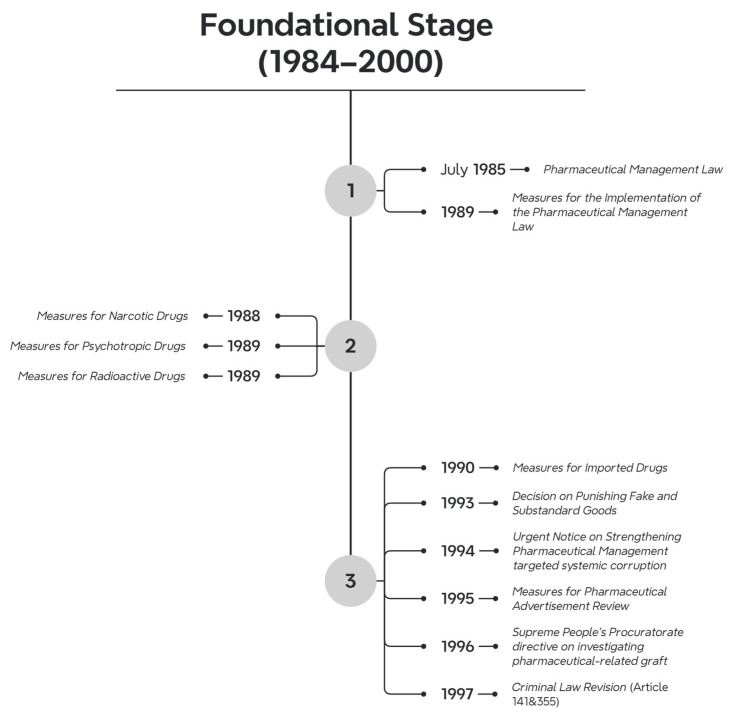
Timeline of legislation during the Foundational stage.

Between 1984 and 2000, a total of 1252 pharmaceutical-related laws and regulations were introduced. The number of documents issued each year was as follows: 4 in 1984, 8 in 1985, 13 in 1986, 11 in 1987, 18 in 1988, 25 in 1989, 27 in 1990, 13 in 1991, 25 in 1992, 22 in 1993, 17 in 1994, 26 in 1995, 26 in 1996, 9 in 1997, 66 in 1998, 620 in 1999, and 322 in 2000. Regarding the hierarchy of legal force, there were 2 laws, 26 administrative regulations, 3 judicial interpretations, and 1229 departmental rules. The issuing bodies were as follows: 2 documents issued by the Standing Committee of the National People’s Congress (NPC), 7 by the State Council, 2 by the Supreme People’s Court, 2 by the Supreme People’s Procuratorate, 327 by various departments of the State Council, 2 by the Communist Party’s central departments, and 950 by other institutions. In terms of validity, 1066 documents are still in effect, 181 have expired, and 5 have been amended. It is worth noting that there was a surge in pharmaceutical-related legislation in 1999, primarily due to the establishment of the NMPA in 1998. Following this, the regulations governing drug management were comprehensively revised under the leadership of this regulatory body. The dominant law of this period was the Pharmaceutical Management Law of 1984 (Figure 4).

#### 3.2.3. Deepening Reform Stage (2001–2018)

**Figure 5:**
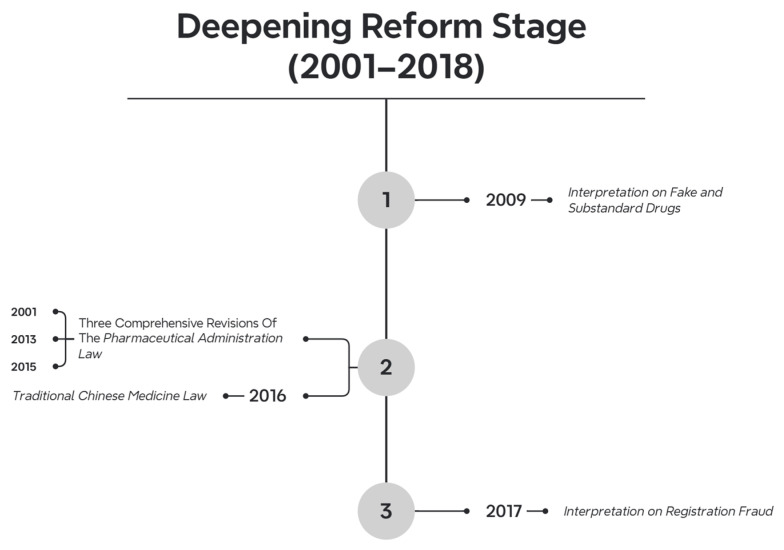
Timeline of legislation during the Deepening Reform stage.

Between 2001 and 2018, a total of 5672 pharmaceutical-related laws and regulations were introduced. The number of documents issued each year was as follows: 407 in 2001, 304 in 2002, 309 in 2003, 253 in 2004, 227 in 2005, 241 in 2006, 248 in 2007, 205 in 2008, 272 in 2009, 291 in 2010, 351 in 2011, 318 in 2012, 340 in 2013, 298 in 2014, 389 in 2015, 462 in 2016, 462 in 2017, and 295 in 2018. Regarding the hierarchy of legal force, there were 8 laws, 37 administrative regulations, 13 judicial interpretations, 5592 departmental rules, 1 military regulation, 17 internal Party regulations, and 6 industry standards. The issuing bodies included the following: 8 by the NPC Standing Committee, 18 by the State Council, 23 by the Supreme People’s Court, 10 by the Supreme People’s Procuratorate, 4784 by various State Council departments, 12 by the Central Military Commission, 26 by Party central departments, and 983 by other institutions. In terms of validity, 5074 documents are still in effect, 548 have expired, 43 have been amended, and 7 are partially expired. This period is characterized by a surge in legislative output, with the dominant law being the Pharmaceutical Management Law of 2001 (Figure 5).

#### 3.2.4. Full Lifecycle Regulation Stage (2019–Present)

**Figure 6:**
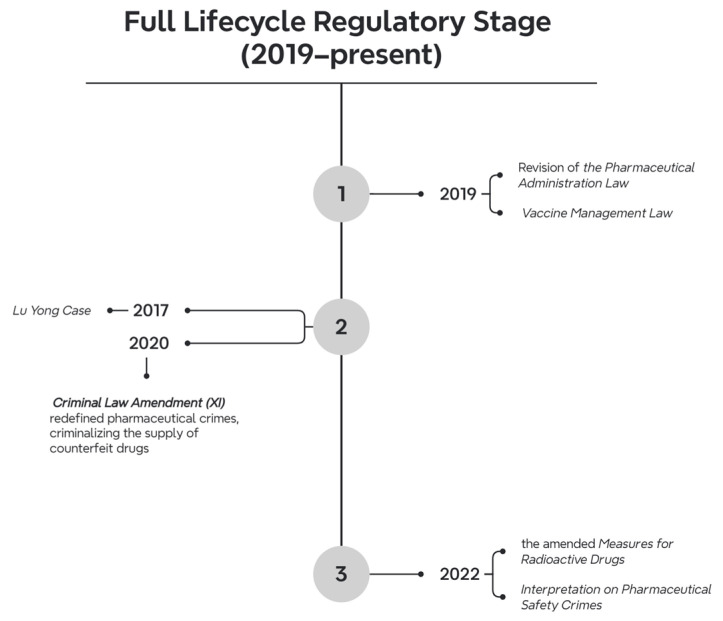
Timeline of legislation during the full Lifecycle Regulation stage.

Since 2019, a total of 740 pharmaceutical-related laws and regulations have been introduced. The number of documents issued each year was as follows: 123 in 2019, 98 in 2020, 104 in 2021, 100 in 2022, 127 in 2023, 171 in 2024, and 17 in 2025. In terms of the hierarchy of legal force, there was 1 law, 13 administrative regulations, 19 judicial interpretations, 706 departmental rules, 1 internal Party regulation, and 1 military regulation. The issuing bodies were as follows: 1 by the NPC Standing Committee, 6 by the State Council, 10 by the Supreme People’s Court, 12 by the Supreme People’s Procuratorate, 176 by various State Council departments, 4 by the Central Military Commission, 2 by Party central departments, and 616 by other institutions. Regarding validity, 719 documents are still in effect, 10 have expired, 7 have been amended, 3 have not yet been implemented, and 1 has partially expired. The dominant legal regulation of this period is the Pharmaceutical Management Law of 2019 (Figure 6).

### 3.3. Content Analysis of Legal and Regulatory Documents Across Different Stages

Drawing on the statistical data presented in Section 3.2, this section conducts a thematic analysis of legislative priorities and regulatory innovations across China’s four developmental stages, revealing how the legal frameworks evolved to address sector-specific challenges and align with socio-economic transformations.

#### 3.3.1. Emergence Stage: Fragmented Governance and Reactive Control

During the pre-reform era (1949–1978), China’s pharmaceutical regulatory framework operated under a decentralized administrative model, relying primarily on ad hoc directives issued by government departments rather than formal legislation. The absence of codified laws resulted in inconsistent enforcement and localized governance, particularly evident in efforts to control narcotics and standardize production. With the advent of economic reforms in 1978, the demand for pharmaceuticals gradually grew, and the pharmaceutical industry began to develop. The government recognized the need to regulate the industry through legislation. Initially, the state initiated a tentative transition toward structured regulation through limited administrative and criminal legislation.

The widespread medical use of narcotics such as morphine and dextromethorphan in the late 1970s precipitated their illegal trafficking and misuse, prompting urgent regulatory action [48]. In September 1978, the State Council promulgated the Regulations on Narcotic Drugs, centralizing the oversight of production, supply, and medical usage. This was followed by the Detailed Rules for Implementation in February 1979, which established licensing protocols and usage restrictions. Subsequent measures in 1982 and 1983—including the Measures for Narcotic Drug Production and Measures for Domestic Transportation of Narcotic Drugs—further tightened controls over manufacturing and distribution channels. Concurrently, efforts to address unregulated pharmaceutical production emerged through the 1979 Regulations on Pharmaceutical Testing Laboratories, mandating quality inspections, and the 1980 Implementation Rules for Pharmaceutical Factory Rectification, which standardized factory conditions and spatial layouts to eliminate illicit operations.

Criminal law also began to intersect with pharmaceutical governance during this period. Article 164 of the 1979 Criminal Law explicitly criminalized the manufacture and sale of counterfeit drugs, marking an early legal deterrent against pharmaceutical fraud. However, enforcement remained uneven due to the fragmented jurisdictional oversight and reliance on departmental rules, which constituted 92.6% of the 27 regulations enacted between 1949 and 1983.

While these measures laid a rudimentary foundation for drug safety, the era was characterized by reactive governance focused on crisis containment rather than proactive risk management. Regulatory priorities centered narrowly on narcotics control and production-stage inspections, neglecting post-market surveillance and innovation incentives. This fragmented approach, compounded by the dominance of non-binding administrative decrees, underscored systemic limitations in achieving cohesive oversight—a challenge that subsequent reforms would seek to address.

#### 3.3.2. Foundational Stage: Codification and Systemic Governance

The rapid expansion of China’s pharmaceutical market during the 1980s, driven by socio-economic liberalization, exposed critical vulnerabilities in drug quality and regulatory oversight [49]. The proliferation of substandard and counterfeit medicines, coupled with regulatory blind spots in distribution channels, posed significant threats to public health, requiring urgent legislative intervention to safeguard public health. This period witnessed the establishment of China’s first comprehensive pharmaceutical legal framework, transitioning from fragmented administrative decrees to codified governance.

The cornerstone of this era was the enactment of the Pharmaceutical Management Law in July 1985, which systematically regulated production, distribution, quality control, and institutional supervision. The law’s scope extended to packaging standards, medical unit responsibilities, and advertising practices, marking a paradigm shift toward centralized oversight. Its implementation was bolstered by the 1989 Measures for the Implementation of the Pharmaceutical Management Law, issued by the Ministry of Health, which operationalized the procedural details for licensing and inspections. Concurrently, the State Council promulgated specialized regulations targeting high-risk drug categories: the Measures for Narcotic Drugs (1988), Measures for Psychotropic Drugs (1989), and Measures for Radioactive Drugs (1989), which imposed stringent controls on production, distribution, and transportation. Supplementary rules, such as the Measures for Imported Drugs (1990) and Measures for Pharmaceutical Advertisement Review (1995), addressed emerging challenges in globalized trade and commercial ethics.

Criminal justice reforms paralleled administrative advancements. The 1993 NPC Decision on Punishing Fake and Substandard Goods introduced a critical legal distinction between counterfeit and substandard drugs, with the former being punishable by death—a stark escalation reflecting heightened public health imperatives. The 1994 Urgent Notice on Strengthening Pharmaceutical Management targeted systemic corruption, mandating crackdowns on bribery in procurement and chaotic market practices. Judicial authorities further reinforced accountability through the 1996 Supreme People’s Procuratorate directive on investigating pharmaceutical-related graft and the 1997 Criminal Law Revision, which codified separate offenses for producing counterfeit drugs (Article 141) and illegally supplying narcotics (Article 355).

While these reforms established a robust administrative criminal governance framework, its implementation revealed structural tensions. The predominance of departmental rules (98.2% of 1252 regulations enacted during 1984–2000) perpetuated their fragmented enforcement, particularly in rural regions. Moreover, the focus on ex post punitive measures—exemplified by the death penalty provisions—overshadowed preventive mechanisms such as pharmacovigilance or consumer education. Nevertheless, this phase laid indispensable groundwork for subsequent reforms, bridging legislative voids and aligning China’s pharmaceutical governance with nascent international norms.

#### 3.3.3. Deepening Reform Stage: Post-Scandal Centralization and Systemic Overhaul

The early 21st century witnessed a paradoxical dynamic in China’s pharmaceutical landscape: while market liberalization catalyzed industrial growth, it simultaneously amplified systemic risks, culminating in a series of catastrophic drug safety crises. High-profile incidents—including the Xinfu case (2006) involving adulterated antibiotics [50]; the Shanxi vaccine scandal (2010), exposing falsified storage records [51]; and the Changsheng vaccine crisis (2018), revealing substandard pediatric vaccines—severely eroded public trust, exposing structural flaws in the regulatory system, and the reputation of the drug regulatory authorities faced a significant public crisis [52]. These crises precipitated a transformative legislative response, characterized by three comprehensive revisions of the Pharmaceutical Administration Law (2001, 2013, 2015) and parallel judicial reforms to address evolving criminal complexities.

The 2001 Amendment marked a watershed, abolishing decentralized local quality standards in favor of unified national norms and introducing mandatory GMP/GSP certifications to enforce production and distribution compliance. This revision streamlined approval processes, classified prescription and non-prescription drugs, and escalated penalties for violations—measures aimed at curbing counterfeit proliferation. Subsequent amendments further modernized governance: the 2013 Revision delegated the oversight of entrusted production to provincial authorities, while the 2015 Amendment transitioned from pre-approval licensing to post-market supervision, aligning with market-driven pricing reforms that replaced state-controlled drug pricing. Complementary updates to the Regulations for Implementation of the Pharmaceutical Administration Law reinforced these structural shifts, culminating in the 2016 Traditional Chinese Medicine Law to integrate heritage practices into standardized regulatory frameworks.

Judicial institutions paralleled legislative advancements through interpretative clarifications. The Supreme People’s Court and Procuratorate issued multiple directives—notably the 2009 Interpretation on Fake and Substandard Drugs and 2017 Interpretation on Registration Fraud—to delineate criminal liabilities for pharmaceutical crimes. These included aggravated penalties for using counterfeit drugs in illegal medical practices (2008) and criminalizing data fabrication in drug approvals (2017). Article 141 of the revised Criminal Law further distinguished between counterfeit and substandard drugs, while Article 355 expanded on the liability for illegal narcotics distribution.

Despite these advancements, the era revealed critical tensions. Rapid legislative output (5672 regulations enacted between 2001 and 2018) strained enforcement capacities, particularly in rural regions lacking regulatory infrastructure. The emphasis on ex post punitive measures—evidenced by 462 pharmaceutical-related convictions in 2016 alone—overshadowed preventive mechanisms such as pharmacovigilance or public education. Moreover, the 2015 market pricing reform, while enhancing flexibility, inadvertently exacerbated affordability challenges for essential medicines. Nevertheless, this phase solidified China’s transition from reactive crisis management to proactive institutional governance, laying essential groundwork for the lifecycle regulation paradigm that would emerge post-2019.

#### 3.3.4. Full Lifecycle Regulation Stage: Integrated Governance and Global Alignment

As the domestic pharmaceutical market becomes increasingly complex and regulatory standards converge globally, China has been compelled to further implement the “Healthy China” strategy. In response to the public health crisis triggered by the 2018 Changchun Changsheng vaccine incident, a paradigm shift in China’s governance model has been driven, further standardizing and strengthening drug regulations. In 2019, the Pharmaceutical Administration Law underwent its most comprehensive revision since its inception, transitioning from fragmented administrative mandates to a holistic, risk-proportionate lifecycle framework. This revision, structured across 12 chapters and 155 articles, institutionalized four pillars of modern regulation: (1) risk management as a guiding principle, emphasizing pre-emptive hazard identification; (2) dynamic quality oversight, replacing static GMP/GSP certifications with real-time inspections and pharmacovigilance systems; (3) innovation facilitation, exemplified by conditional approvals for breakthrough therapies (e.g., CAR-T) and priority review pathways that reduced approval timelines by 40% (2020–2023); and (4) social co-governance, integrating public participation through enhanced transparency mechanisms.

Concurrently, the 2019 Vaccine Management Law consolidated previously dispersed legislation into a unified regime, imposing stringent controls across R&D, production, distribution, and vaccination. This law’s “strictest-in-history” provisions—mandating real-time lot tracking and emergency recall protocols—directly responded to the 2016 Changsheng scandal, restoring public confidence post-crisis. Further reforms in 2022 decentralized regulatory authority, as seen in the amended Measures for Radioactive Drugs, which delegated approval powers to specialized enterprises from central to provincial levels, enhancing administrative efficiency.

Judicial and societal dynamics mirrored legislative advancements. The 2020 Criminal Law Amendment (XI) redefined pharmaceutical crimes, criminalizing the supply of counterfeit drugs and introducing the novel offense of “obstructing drug management”. This amendment resolved ambiguities in cross-border drug procurement—epitomized by the Lu Yong case (2017) [53] and its cinematic portrayal in Dying to Survive (2018)—by decriminalizing personal imports while penalizing commercial resale. In 2022, the Supreme People’s Court and Procuratorate issued the Interpretation on Pharmaceutical Safety Crimes, delineating thresholds for criminal liability in cases of data fraud or negligence.

Despite these strides, challenges persist. The 15% increase in API international certifications (2021–2023) underscores improved compliance, yet dependence on imported intermediates exposes supply chain vulnerabilities. Rural–urban disparities in inspection capacity and the ethical tensions of conditional approvals—highlighted by the 2021 withdrawal of a gene therapy due to incomplete safety data—reveal systemic fissures. Nevertheless, this stage epitomizes China’s strategic pivot from command-control rigidity to agile, science-driven governance, positioning its regulatory framework as a hybrid model with lessons for global health governance—particularly in balancing innovation imperatives with pandemic-era safety demands.

## 4. Discussion

The transition of China’s pharmaceutical regulatory framework from a “command-control” model to “lifecycle regulation” reflects not only the modernization of governance concepts but also the complexity of balancing drug innovation, safety assurance, and public health imperatives. The following sections critically evaluate the achievements, challenges, and policy implications of this transformation.

### 4.1. Achievements

China’s transition to a lifecycle regulatory framework has yielded significant progress in drug safety, innovation facilitation, and accessibility. These achievements reflect the strategic alignment of legislative rigor, technological integration, and policy adaptability, positioning China as a pivotal player in global pharmaceutical governance.

#### 4.1.1. Strengthened Drug Safety Governance

China’s regulatory reforms have institutionalized a multi-layered safety framework that integrates pre-market risk assessments, real-time monitoring, and post-market accountability. The implementation of a nationwide drug traceability system represents the cornerstone of these efforts. By leveraging blockchain technology and IoT-enabled tracking (e.g., QR codes), this system ensures full visibility across the pharmaceutical supply chain. For example, Henan Province’s Eagle Eye Project—a pilot initiative combining AI-driven video analytics and big data—reduced inspection times by 60% while improving anomaly detection accuracy by 35% (2021–2023) [54]. Such innovations align with the U.S. FDA’s Drug Supply Chain Security Act (DSCSA) [55], but are uniquely tailored to China’s scale and administrative complexity.

Post-market surveillance mechanisms have also been fortified. In 2023, China’s National Adverse Drug Reaction (ADR) Monitoring Network processed 2.419 million reports, achieving 98.5% county-level coverage—a 12% increase from 2020 [56]. High-risk drugs, such as biologics and gene therapies, undergo mandatory periodic safety reviews, with recalls stratified into three tiers: Level I (immediate threat, 14.29%), Level II (significant risk, 44.81%), and Level III (minor risk, 40.91%) [57]. These measures mirror the EU’s pharmacovigilance framework but incorporate localized enforcement protocols to address regional disparities.

#### 4.1.2. Accelerated Pharmaceutical Innovation

Regulatory modernization has catalyzed R&D efficiency without compromising safety. The introduction of priority review pathways for breakthrough therapies—such as CAR-T cell treatments and mRNA vaccines—shortened median approval timelines from 24 to 14 months (2020–2023) [58]. A notable case is the expedited approval of sintilimab, a domestically developed PD-1 inhibitor, which received market authorization in 2021 after demonstrating superior efficacy in Phase III trials [59]. This approach parallels the U.S. FDA’s Breakthrough Therapy Designation [60], but emphasizes domestic innovation through fiscal incentives, such as tax rebates for R&D investments exceeding CNY 50 million [61].

Furthermore, China’s 2017 accession to the International Council for Harmonisation (ICH) enabled the mutual recognition of clinical trial data, reducing redundant studies and accelerating global drug launches [62]. The adoption of AI-powered review tools, such as the Intelligent Regulatory System (IRS), further enhanced efficiency, achieving a 99.9% review completion rate without delays [26]—surpassing the EMA’s 94% benchmark.

#### 4.1.3. Enhanced Equitable Access

China has transitioned from drug scarcity to equitable access through targeted policy interventions. The Volume-Based Procurement (VBP) program—a bulk-purchasing mechanism—reduced prices for 52 essential medicines by an average of 53% in 2022, benefiting over 40 million patients annually [63]. This model echoes the UK’s NHS cost-containment strategies [64], but integrates tiered pricing to accommodate regional economic disparities.

To address rural–urban access gaps, a National Drug Shortage Monitoring Platform was launched in 2020, integrating data from 3200 hospitals and 18,000 pharmacies. This system facilitated the redistribution of 15,000 drug batches to underserved regions in 2023 alone [65]. Additionally, the strategic stockpiling of critical APIs and vaccines bolstered supply chain resilience during the COVID-19 pandemic, ensuring uninterrupted access to life-saving therapies.

### 4.2. Challenges

This section discusses the challenges in China’s drug regulation, including regional enforcement disparities, the tension between conditional approval and risk mitigation, the dependency on global supply chains, and the competition of multiple interests. It emphasizes the need for improved management strategies.

#### 4.2.1. Imbalance in Regulatory Enforcement Capacity

A critical challenge lies in the geographic and administrative disparities in regulatory enforcement. In economically advanced regions such as Beijing, Shanghai, and Guangdong, drug oversight is robust, characterized by stringent quality monitoring and streamlined approval processes. Conversely, under-resourced areas in central and western China face systemic deficiencies, including inadequate technical infrastructure and limited personnel training, which hinder the effective detection and deterrence of counterfeit and substandard drugs. The urban–rural divide further exacerbates accessibility and safety issues. High logistics costs and prolonged distribution cycles result in frequent shortages, while limited regulatory oversight allows unlicensed pharmacies to proliferate. Compounding these issues, rural healthcare providers often lack pharmacovigilance training, leading to widespread antibiotic misuse.

#### 4.2.2. Risk–Benefit Trade-Offs in Conditional Approvals

Conditional approval often relies on early clinical data or limited trial results, meaning that the long-term safety and efficacy of a drug may not be fully established. As a result, the potential risks may not be adequately assessed, increasing risks to the public and patients once the drug enters the market. Regulatory bodies typically impose risk mitigation measures, such as limiting the drug’s use or requiring additional trials. However, balancing the urgent need for the drug with controlling its potential risks is a major challenge. While conditional approval accelerates market entry to address public health emergencies, it may lead to insufficient safety evaluations, especially for drugs without long-term trial data. Regulatory agencies address this by requiring post-market trials, enhanced monitoring of adverse reactions, or restricting indications. However, practical challenges like limited resources can hinder these efforts, leaving some drugs on the market with unassessed risks. For example, in China, the National Medical Products Administration (NMPA) has granted conditional approvals for some innovative drugs, but their long-term safety remains unverified [66]. A key example is the emergency use authorization of COVID-19 vaccines [67]. Despite expedited approval, the lack of comprehensive safety data led to enhanced post-market monitoring and usage restrictions.

#### 4.2.3. Over-Reliance on Global Pharmaceutical Supply Chains

China’s dependency on imported active pharmaceutical ingredients (APIs) and advanced biotechnologies poses systemic risks. Over 70% of oncology drugs and 85% of vaccine adjuvants rely on foreign suppliers, primarily from the U.S., Europe, and India [68]. Geopolitical disruptions, such as the 2022 Russia–Ukraine conflict, triggered a 28% surge in API procurement costs and delayed production for 32 domestic manufacturers [69]. During the COVID-19 pandemic, global logistics bottlenecks exacerbated the shortages of critical intermediates, delaying 25% of vaccine production schedules [70].

Domestic innovation gaps further perpetuate this dependency. Despite progress in generics, China contributes only 18% of global novel drug patents, lagging behind the U.S. (48%) and EU (27%) [71]. High-tech sectors, including mRNA vaccines and gene therapies, remain reliant on imported equipment and expertise, with 80% of biopharmaceutical R&D partnerships involving foreign firms. This reliance leaves China vulnerable to trade policy shifts, as seen in the 2021 U.S. export controls on synthetic biology tools, which stalled 15% of domestic biotech projects.

#### 4.2.4. The Competition of Multiple Interests

The formulation of China’s drug management laws and regulations is typically initiated by the National Medical Products Administration (NMPA) or its relevant departments. The drafting process involves not only technical discussions but also considerations of various social needs, economic interests, and the political environment. The entire process, from drafting to formal enactment, is often government-led, with political considerations playing a significant role in driving legal revisions. During the revision of the drug management law, relevant departments such as the National Health Commission and the Ministry of Finance are involved, reflecting broad political collaboration.

During the public consultation phase, the government generally seeks input from various sectors of society. Stakeholders in the pharmaceutical industry, including pharmaceutical companies, academic organizations, patient groups, and even local governments, all participate in this process. Additionally, the opinions of the public, scholars, and experts are also highly valuable. Throughout the revision process, the voices of different interest groups directly influence the provisions of the law. For example, the accelerated drug review process may be driven by the pharmaceutical industry, while regulatory strengthening provisions may gain support from the government and consumer protection organizations. Furthermore, the interaction between domestic pharmaceutical companies and foreign entities in the market is another key factor in updating drug management regulations. As many foreign pharmaceutical companies enter the Chinese market, China needs to adopt policies that attract foreign investment, align certification standards with international practices, and balance the competition between domestic and foreign companies.

This competition between multiple interests is similar to the situation in the United States and the European Union. In the U.S., the formulation of drug management regulations is often influenced by partisan politics and industry interests, especially those of large pharmaceutical companies. Public hearings and the process of soliciting opinions are key stages in this process. Under the guidance of the FDA, drafts typically undergo rigorous public consultation, where stakeholders can participate directly. In the EU, political compromises are necessary to balance the interests of member states. Compared to China, both the U.S. and the EU have a higher level of transparency in public consultation, with greater openness in the competition between interests.

### 4.3. Implications

Firstly, balancing regional disparities in drug resources is a critical aspect of achieving equitable drug management. To address the unequal distribution of resources between regions, particularly between urban and rural areas, China could implement region-specific policies that focus on providing preferential support to underserved areas, ensuring greater access to essential medicines. For instance, the United States’ 340B Drug Discount Program offers discounted drugs to safety-net hospitals in low-income areas, making medications more affordable for vulnerable populations. A similar approach could be adopted in China by enhancing rural healthcare infrastructure, particularly by establishing more drug distribution centers and healthcare facilities to ensure timely medication delivery. Additionally, the development of digital platforms could enhance the efficiency and transparency of drug distribution. The European Medicines Agency facilitates the coordination of drug supply across member states, which helps to bridge regional gaps in drug availability. China could take inspiration from this model by encouraging the growth of local pharmaceutical companies and improving cross-regional drug allocation. Moreover, integrating telemedicine with drug delivery services would ensure that patients in remote areas receive both pharmaceutical advice and medications in a timely manner, further improving access to healthcare.

Secondly, improving domestic drug innovation is a priority. The government must increase investment in drug research and development, particularly in areas such as biopharmaceuticals, gene therapy, and antibody drugs. Creating dedicated funds or incentive programs would encourage pharmaceutical companies to pursue high-risk, high-reward innovations. For instance, the European Union’s Horizon Europe program provides funding for drug development, focusing on novel therapies, precision medicine, and innovative vaccines. The European Investment Bank (EIB) also offers loans and financial support to help pharmaceutical companies complete their drug development. Additionally, fostering collaboration between public research institutions and pharmaceutical companies is crucial. Strengthening partnerships between public institutions (such as research academies and medical centers) and pharmaceutical companies could help to translate foundational research into drug innovations. For example, the JLABS (Johnson & Johnson Innovation Lab) initiative in the United States provides biotech startups with access to research resources, lab space, and technical support, collaborating with Johnson & Johnson to accelerate product development and commercialization.

Thirdly, enhancing phased drug regulation and risk management is essential. Post-market surveillance should be further refined by dividing it into distinct phases, each with varying levels of risk assessment. Continuous monitoring should be required, with pharmaceutical companies regularly updating the data for each phase. The European Union employs the “EudraVigilance” system to monitor adverse drug reactions, allowing healthcare professionals, patients, and the public to report side effects. All data are uploaded to a centralized platform for analysis. Similarly, China could strengthen its post-market surveillance system by adopting a similar approach, ensuring continuous safety assessments of drugs after they enter the market.

In summary, achieving balanced drug access, fostering domestic drug innovation, and enhancing phased drug regulation and risk management are critical steps toward improving China’s drug management system. By learning from successful international models, China can strengthen its regulatory framework, ensuring equitable access to safe and effective medicines for all its citizens. The strength of this study lies in its analysis of pharmaceutical regulatory laws and regulations, offering a clearer understanding of the advantages of China’s current regulatory model, as well as the areas that still need improvement. However, while the study offers an in-depth review of these laws, its primary focus is on assessing the existing regulations rather than addressing the specific challenges encountered in the practical operations of the pharmaceutical industry. For instance, it does not examine the practical difficulties or obstacles faced in regulating drug production, distribution, and sales. As a result, this study provides limited insight into these practical issues, and further research is needed to fill these gaps, enabling a more comprehensive understanding and resolution of the challenges that arise in drug regulation.

## 5. Conclusions

Transition from “Command-Control” to “Lifecycle Regulation”: China’s drug regulatory system has undergone a significant transformation, shifting from a “command-control” model to a “lifecycle regulation” framework. This shift represents a major evolution in pharmaceutical governance, aligning domestic policies with global trends while addressing both safety and innovation challenges.

Adoption of the Lifecycle Regulation Model: The adoption of the lifecycle regulation model, which includes pre-market evaluation, post-market monitoring, and real-time pharmacovigilance, has improved drug safety, streamlined approval processes, and promoted innovation, especially in the biopharmaceutical sector. However, challenges persist, including regional disparities in regulatory enforcement, the complex balance between conditional approvals and risk mitigation, and an over-reliance on global supply chains for critical medications.

Potential of Lifecycle Regulation in Balancing Safety, Innovation, and Accessibility: The lifecycle regulation model holds significant potential in balancing safety, innovation, and accessibility. Moving forward, addressing enforcement disparities, particularly between urban and rural areas, and further strengthening domestic innovation capabilities will be essential. Additionally, leveraging digital technologies and promoting cross-regional regulatory coordination could help to alleviate some of the current challenges. By continuing to refine its regulatory framework, China could become a model for other developing countries seeking to balance drug safety, rapid innovation, and public health security.

Future Research on Drug Management Violations and Crimes: Future empirical research on recent drug management violations and crimes in China could uncover the common issues in pharmaceutical practices and propose solutions. This research could provide valuable insights into the governance of drug management violations, offering lessons for other countries.

## Figures and Tables

**Table 1:** Inter-coder reliability tests.

Component	Value/Description	Notes
Test Type	Inter-coder reliability	Ensured consistency in the thematic coding of legal texts.
Statistical Metric	Cohen’s Kappa coefficient (κ)	Measures in agreement beyond chance (Landis & Koch [47]).
Number of Coders	2	Independent coders with expertise in pharmaceutical law and policy.
Sample Size	10 texts (20% of total n = 51)	Randomly selected subset of legal documents.
Kappa Score	κ = 0.82	Indicates substantial agreement (Landis & Koch criteria: 0.61–0.80).
Key Discrepancies	Terminology ambiguity	e.g., “dynamic inspections” vs. “risk-based evaluations”.
Resolution Process	Iterative discussion + legislative intent	Ambiguities resolved via official commentaries and expert consultation.

## Data Availability

The data used in this study consist of legally binding texts from Chinese laws and regulations, including statutes, administrative rules, and policy documents. These legal texts are publicly available and can be accessed through official government platforms, such as PKULAW (https://www.pkulaw.com/, accessed date: 24 November 2024), the U.S. FDA Regulations (https://www.fda.gov/regulatory-information/fda-rules-and-regulations, accessed date: 24 November 2024), and the EU EMA Guidelines (https://www.ema.europa.eu/en, accessed date: 24 November 2024), where no new data were created or where data were unavailable due to privacy or ethical restrictions. The data used in this study are accessible through the above-mentioned public sources, and further information can be provided upon request from the corresponding author.
